# Editorial: Adult Neurogenesis: Beyond Rats and Mice

**DOI:** 10.3389/fnins.2018.00904

**Published:** 2018-12-04

**Authors:** Luca Bonfanti, Irmgard Amrein

**Affiliations:** ^1^Neuroscience Institute Cavalieri Ottolenghi, Orbassano, Italy; ^2^Department of Veterinary Sciences, University of Turin, Torino, Italy; ^3^Division of Functional Neuroanatomy, Institute of Anatomy, University of Zurich, Zurich, Switzerland; ^4^Department of Health Sciences and Technology, ETH Zurich, Zurich, Switzerland

**Keywords:** comparative studies, evolution, brain plasticity, adult neurogenesis, brain repair, translation

Most biological tissues routinely replace old cells with new ones. Unlike other tissues, the nervous system–being the most complex biological device found in nature–uniquely maintains most of its neurons throughout life and replaces relatively few. It preserves hotspots where it generates new neurons from resident stem cells during adulthood in a process known as adult neurogenesis, which varies among different species in its features, dynamics, and regulation. In spite of its widespread prevalence in the animal kingdom, the preponderance of studies conducted on a few laboratory rodent species such as rats and mice limits our understanding of the evolution, regulation, and function of adult neurogenesis. The anatomy, complexity and functions of the brain vary greatly in the animal kingdom: striking differences exist from simple bilaterians to humans, and, to a lesser extent, also among mammals. Therefore, both comparative and focused studies on different species will shed more light on the origin, development, and purpose of adult neurogenesis.

Adult neurogenesis was discovered and described by Joseph Altman and Das in rats (Altman and Das, 1965) and has been investigated in many species such as the zebrafish, frog, songbird, mole, mole-rat, vole, bat, fox, dog, dolphin, elephant, shrew, rabbit, monkey, and human. With the development of genetic manipulation techniques, researchers have focused largely on inbred laboratory rodents. While this provides a strong advantage of restricting genetic variation in the group, it also narrows our perspective on adult neurogenesis as a biological phenomenon (Bolker, 2017). Moreover, the rapid development of genetic tools has made *Mus musculus* the species of choice in studying adult neurogenesis. Yet, many unsolved issues and open questions cannot be resolved without the contribution of comparative studies spanning through widely different species. Such issues involve: how did adult neurogenesis evolve, whether our survival depend on adult neurogenesis, what is the link between adult neurogenesis and brain complexity, how do adult neurogenesis and animal behavior influence each other, how does adult neurogenesis contribute to brain plasticity, cognition and, possibly, repair, and how do experimental conditions affect adult neurogenesis.

Studying unconventional species will give us insights into the evolution and function of the brain, strengthening our understanding of the cellular basis of cognition and behavior, thus helping adult neurogenesis to find its place in the puzzle. With this Research Topic we, along with contributors from different areas, tried to answer the open questions and to encourage engaging discussions on the comparative and evolutionary aspects of adult neurogenesis. The diversity in adult neurogenesis indeed spans the *de-novo* formation of the entire adult brain in planaria (Brown and Pearson), neurogenesis in diverse brain areas in fish (Olivera-Pasilio et al.), reptiles (LaDage et al.; Lutterschmidt et al.), and birds (Barkan et al.; Kosubek-Langer et al.) to animals with restricted neurogenic niches such as invertebrates (Beltz and Benton; Simões and Rhiner) and mammals (Taylor et al.; Lévy et al.; Oosthuizen; Wiget et al.). The striking differences do not only concern the sites of occurrence and relative amounts (Brown and Pearson; Lévy et al.; Olivera-Pasilio et al.; Wiget et al.) but also in mechanistic aspects of stem cell biology. Intriguing examples are given by the adult-born neurons generated from the immune system and then traveling to the neurogenic niche via the circulatory system in the crayfish brain (Beltz and Benton; Simões and Rhiner), or the heterogeneity of neoblasts, putative stem cells, in flatworms enabling the regeneration of the entire brain (Brown and Pearson). Yet, the main message from the comparative approach to adult neurogenesis is that the relative exclusive focus on laboratory rodents can result in a bias on how we think about this biological process. For instance, promising neuroprotective treatments developed in rodent models can fail in preclinical trials, and animal models with gyrencephalic brains might be necessary to study the behavior of neuroblasts in large white matter tracts (Taylor et al.). The bias is well-illustrated by the article of Faykoo-Martinez et al.: “species-specific adaptations in brain and behavior are paramount to survival and reproduction in diverse ecological niches and it is naive to think adult neurogenesis escaped these evolutionary pressures. A neuroethological approach to the study of adult neurogenesis is essential for a comprehensive understanding of the phenomenon.” Indeed, interactions of adult neurogenesis with neuroethological traits such as migration and mating behavior in snakes (Lutterschmidt et al.), territoriality in lizards (LaDage et al.), sociality and social interactions in mole-rats, birds, and sheep (Barkan et al.; Lévy et al.; Oosthuizen), or migratory lifestyle in birds (Barkan et al.) are presented here. The complexity of interactions is, to date, more an obstruction than a help in terms of publishability, but as Faykoo-Martinez et al. put it “most of us are guilty of making strong assertions about our data in order to have impact yet this ultimately creates bias in how work is performed, interpreted, and applied.” Such concerns are confirmed by the finding of remarkable reduction of adult neurogenesis in some large-brained, long-living mammals, including humans and dolphins (Sanai et al., 2011; Sorrells et al., 2018), as reviewed and discussed in the article by Parolisi et al. More and more comparative data strongly support the view that adult neurogenesis is maintained in evolution only depending on strict relationships with its functional need(s). E.g., olfactory systems, mostly linked to paleocortical-hippocampal structures, were important in early mammalian evolution working as a reference system for spatial navigation for the location of food sources and mates, then replaced/integrated by the expansion of the isocortex as a “multimodal interface” for behavioral navigation based on vision and audition (Aboitiz and Montiel, 2015; see article by Parolisi et al.). The complex evolutionary aspects of adult neurogenesis role(s) and age-related reduction in mammals are addressed in the contribution by Hans-Peter Lipp. The main message of this opinion article is that no simple explanations can be called upon on such topic, a heavily actual conclusion even 30 years after neural stem cell discovery.

Animal models other than laboratory mice are by no means “out-of-reach” for advanced techniques, and the following examples could encourage and facilitate creative thinking in terms of research questions and how to approach them. Lindsey et al. present a thorough step-by-step protocol for visualizing cell proliferation in the *whole* zebrafish brain in 3 dimension. LaDage et al. used hormonal implants in lizards to study the interaction of testosterone and neurogenesis on territorial behavior. In fish and birds, Neurobiotin or lentivirus can be used to trace and characterize newly born neurons (Kosubek-Langer et al.; Olivera-Pasilio et al.), and Brown and Pearson summarize the single-cell genomic data collected in planaria. Ideally, studies in laboratory rodents and non-conventional animal models can support and foster each other. For example, increased neurogenesis in laboratory mice confers stress resilience mediated by the temporal hippocampus (Anacker et al., 2018). Strikingly, wild rodents, naturally exposed to high stress levels, show more neurogenesis in the temporal hippocampus than the commonly used laboratory mouse C57BL/6 (Wiget et al.). Similarly, Reyes-Aguirre and Lamas identified the mechanism why the mouse retina cannot regenerate after damage, much in contrast to what has been reported in fish (Raymond et al., 2006). Finally, by using meta-analyses and a model to compare the neurodevelopmental sequences of different mammals, Charvet and Finlay try to put in a common time frame the envelopes of hippocampal neurogenesis, in order to interpret them in species with highly different lifespan.

In conclusion, with this Research Topic we strongly assert that adult neurogenesis research cannot rely exclusively on laboratory rodents, as each animal model can only cover certain aspects of the various flavors in which neuronal stem cells and their progeny in the postnatal brain can behave. The papers presented here emphasize the value of “… taking a step back and actually placing our results in a much larger, non-biomedical context, …[helping]… to reduce dogmatic thinking and create a framework for discovery” (Faykoo-Martinez et al.). After all, the failure of many clinical trials based on pre-clinical studies carried out on mice (Lindvall and Kokaia, 2010; Donegà et al., 2013), do confirm the need for investments in comparative medicine (specifically on brain structural plasticity, see La Rosa and Bonfanti, 2018). A comparative view can indeed foster a more careful interpretation of the final impact of the biological process of neurogenesis in brain functioning and animal behavior.

## Author Contributions

All authors listed have made a substantial, direct and intellectual contribution to the work, and approved it for publication.

### Conflict of Interest Statement

The authors declare that the research was conducted in the absence of any commercial or financial relationships that could be construed as a potential conflict of interest.
